# Place du scanner hélicoïdal dans le traumatisme du rocher: à propos de 12 cas au Centre Hospitalier Universitaire Ibn Rochd de Casablanca

**DOI:** 10.11604/pamj.2022.41.72.29902

**Published:** 2022-01-25

**Authors:** Ousmane Traore, Ilias Guindo, Soukaina Wakrim, Aboubacar Sidiki N’Diaye, Mamadou Dembele, Issa Cisse, Moise Dackouo, Daniel Wappa Dembele, Adama Diaman Keita

**Affiliations:** 1 Service de Radiologie 20 Août, Centre Hospitalier Universitaire Ibn Rochd, Casablanca, Maroc,; 2 Faculté de Médecine et d´Odontostomatologie (FMOS), Université des Sciences, des Techniques et des Technologies de Bamako (USTTB), Bamako, Mali,; 3 Service de Radiologie, Clinique Médicale Marie Curie, 37700 Saint-Pierre-des-Corps, France,; 4 Service de Radiologie, Centre Hospitalier Universitaire, Hôpital du Point G, Bamako, Mali

**Keywords:** Rocher, scanner, traumatisme, urgence, Petrous bone, computed tomography, trauma, emergency

## Abstract

Les traumatismes de l´os temporal concernent 14 à 22% des fractures du crâne survenant dans le cadre du traumatisme crânien. Le but de notre étude est de connaitre l´apport du scanner hélicoïdal dans le traumatisme du rocher et illustrer les types de fractures et les lésions associées. C´était une étude rétrospective portant sur 12 cas de traumatisme des rochers dont 10 hommes et 2 femmes sur une période de 14 mois. La moyenne d´âge était de 30 ans, avec un extrême allant de 18 à 42 ans. Le scanner des rochers multicoupes haute résolution sans injection de produit de contraste (PDC), avec des coupes infra-millimétriques de 0,6mm tous les 0,3mm a permis de retrouver les fractures suivantes: 8 cas de fracture transversale extra-labyrinthique; 1 cas de fracture longitudinale extra-labyrinthique; 2 cas de fracture trans-labyrinthique et 1 cas de fracture oblique. Les lésions associées sont dominées par: 5 cas de de lésions ossiculaires; 4 cas d´atteinte de l´os temporal et 2 cas d´atteinte du ganglion géniculé. La tomodensitométrie de haute résolution permet d'affirmer l'existence de la fracture, de décrire l'orientation du trait et de préciser les différentes structures atteintes. Elle peut être réalisée dans l´urgence ou à distance de l´urgence.

## Abstract (en)

Temporal bone injuries occur in 14-22% of skull fractures occurring due to head trauma. The purpose of this study is to understand the role of helical computed tomography in petrous bone trauma and to show the different types of fractures and the associated lesions. We conducted a retrospective study of 12 patients with petrous bone trauma (including 10 men and 2 women) over a period of 14 months. The average age of patients was 30, ranging from 18 to 42 years. High-resolution multi-slice computed tomography of petrous bone without contrast agent injection, with infra-millimeter slices thickness of 0.6mm every 0.3mm, allowed to detect the following fractures: 8 extralabyrinthine transverse fractures; 1 extralabyrinthine longitudinal fracture; 2 translabyrinthine fractures and 1 oblique fracture. The associated lesions were dominated by: 5 ossicular lesions; 4 cases of temporal bone involvement and 2 cases of geniculate ganglion involvement. High-resolution computed tomography can confirm the presence of a fracture, show the orientation of the fracture line and specify the different structures affected. It can be performed for emergency assessment or after a period of observation.

## Introduction

Les traumatismes de l´os temporal concernent 14 à 22% des fractures du crâne survenant dans le cadre du traumatisme crânien. Ils sont d´interprétation difficile du fait de la diversité des formes cliniques et de la complexité de l´anatomie de l´oreille. Les progrès de l´imagerie, notamment en résolution spatiale permettent une meilleure approche des structures les plus fines de l´os temporal. La TDM (tomodensitométrie) reste l´examen de référence dans la caractérisation des lésions du rocher. L´imagerie par résonnance magnétique (IRM) est un complément indispensable pour l´étude du labyrinthe et du nerf facial [1, 2]. Le but de notre travail est de connaitre l´apport du scanner hélicoïdal dans le traumatisme du rocher et illustrer les types de fractures intéressant le rocher avec les lésions associées.

## Méthodes

Il s´agissait d´une étude rétrospective descriptive de 14 mois portant sur 12 examens TDM des traumatismes des rochers colligés au service de radiologie de l´Hôpital 20 août 1953- CHU de Casablanca. Ont été inclus dans cette étude, les patients adressés pour TDM des rochers dans un contexte d´urgence post-traumatique et à distance des urgences. La collecte des données a été faite à partir des dossiers, des bulletins et comptes rendus des examens du scanner. Ils ont permis de recueillir les informations sur l´âge, le sexe, la technique utilisée, les types de fractures observées et les lésions associées. Les examens ont été réalisés avec un scanner de marque GE (général électrique) 16 barrettes sans injection de produit de contraste portant sur les rochers de haute résolution avec des coupes infra-millimétriques de 0.6mm tous les 0.3mm avec de fenêtre de lecture large (4000UH) centrée sur 400 à 800 UH. Une reconstruction axiale (plan parallèle au plan du canal semi-circulaire latéral) et une reconstruction coronale (perpendiculaire au plan axial).

## Résultats

**Figure 1:**
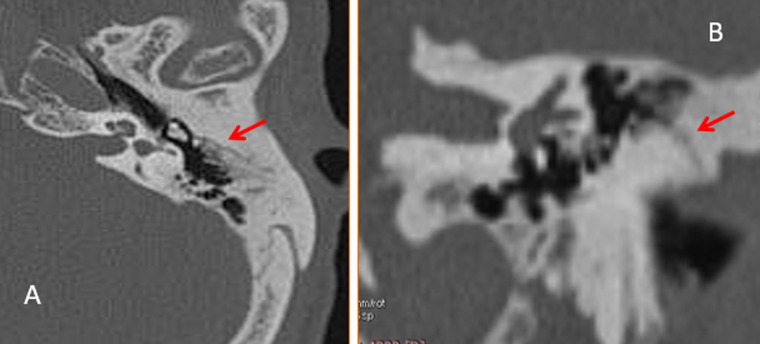
tomodensitométrie des rochers avec reconstruction axiale (A) et coronale (B) montrant les fractures transversales extra-labyrinthiques

**Figure 2:**
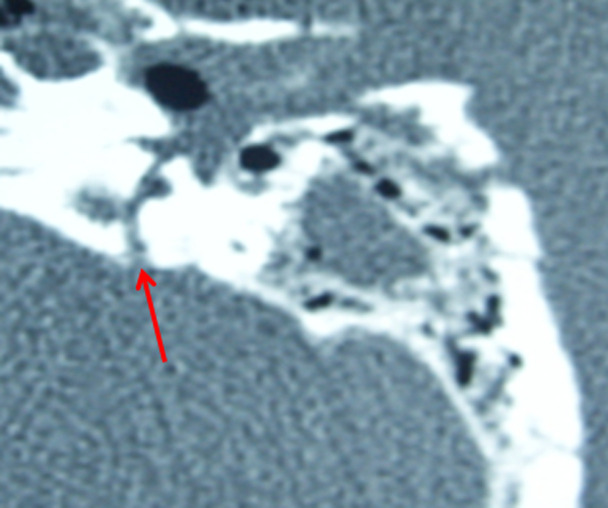
tomodensitométrie des rochers avec reconstruction axiale montrant les fractures transversales trans-labyrinthiques

**Figure 3:**
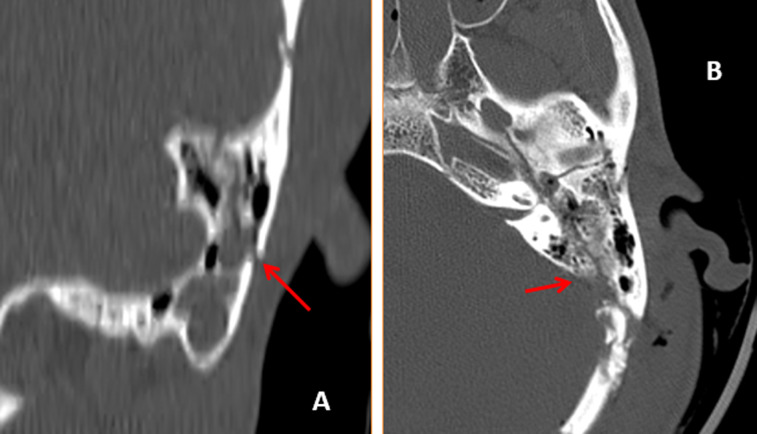
tomodensitométrie des rochers avec reconstruction axiale (A) et coronale (B) montrant les fractures longitudinales et obliques extra-labyrinthiques

Il s´agissait de 10 hommes et 2 femmes. La moyenne d´âge est de 30 ans (extrêmes: 18 à 42 ans). Le motif d´exploration en scanner était le traumatisme à 100% des cas avec une répartition en traumatisme post AVP (accident de la voie publique) chez 7 patients, le traumatisme post coup et blessure volontaire (CBV) chez 3 patients et enfin 2 patients étaient adressé pour traumatisme après chute (Tableau 1). On a retrouvé également les signes cliniques associées entre autre nous avons la paralysie faciale (3 cas), otorragie (6 cas), surdité (3 cas), vertige (5 cas) et nystagmus horizontal (1 cas) (Tableau 2). En TDM, les lésions fracturelles retrouvés étaient (Tableau 3): 8 cas de fracture transversale extra-labyrinthique (Figure 1); 1 cas de fracture longitudinale extra-labyrinthique; 2 cas de fracture translabyrinthique (Figure 2) et 1 cas de fracture oblique (Figure 3). Les lésions associées retrouvées après l´exploration des rochers à la TDM sont (Tableau 2): 5 cas de lésions ossiculaires (2cas de luxation uncudo-maléaire, 1 cas de luxation uncudo-stapèdienne et 2 cas de luxation de l´étrier). L´atteinte de l´os temporal est retrouvée dans 4 cas; 2 cas de l´atteinte du ganglion géniculé; 1 cas de l´atteinte de la 2^e^ et 3^e^ portion du canal du nerf facial et 1 cas de l´atteinte du canal carotidien.

**Tableau 1:** répartition des patients selon les étiologies

Etiologies	Patients	Pourcentage
Traumatisme post AVP	7	28,33%
Traumatisme post CBV	3	25%
Chute	2	16,67%

**Tableau 2:** répartition des patients selon les types de fractures et les lésions associées

Types de fractures	Patients/Pourcentage
Fracture transversale extra-labyrinthique	66,7%
Fracture longitudinale extra-labyrinthique	8,33%
Fracture trans-labyrinthique	16,67%
Fracture oblique	8.33 %
**Les lésions associées**	**Patients**
Luxation uncudo-maléaire	2
Luxation uncudo-stapèdienne	1
Luxation de l´étrier	2
Atteinte de l´os temporal	4
Atteinte du ganglion géniculé	2
Atteinte du 2ème et 3ème portion du canal du nerf facial	1
Atteinte du canal carotidien	1

**Tableau 3:** répartition des patients selon les signes cliniques

Patients	Signes cliniques
Patient 1	Surdité + vertiges
Patient 2	Hypoacousie + vertiges + nausées
Patient 3	Otorragie + surdité
Patiente 4	Surdité + otorragie + nausée
Patient 5	Vertiges + trouble de l´équilibre
Patient 6	Otorragie + hypoacousie
Patiente 7	Otorragie + paralysie faciale
Patient 8	Paralysie faciale + vertiges
Patient 9	Otorragie + vertiges + vomissement
Patient 10	Otorragie + nausée
Patient 11	Vertiges + nystagmus
Patient 12	Paralysie faciale + nausée + vomissement

## Discussion

Au cours de l´exploration scanographique dans les traumatismes cranio-encéphaliques, ces lésions traumatiques du rocher étaient retrouvés, mais le scanner cérébral avec reconstructions en coupes fines en fenêtres osseuses sur le rocher est insuffisant. L´évaluation clinique des fractures du rocher passe souvent au second plan d´où le rôle essentiel du radiologue dans le bilan lésionnel avec une description minutieuse des structures atteintes en fin d´évaluer des séquelles et des complications potentielles Jusqu´à 1/3 des fractures du rocher ne sont pas détectées à la TDM initiale avec un bilan lésionnel imprécis. D´où la nécessité de réaliser un scanner des rochers dédié en haute resolution. Nous avons exploré tous les patients à la TDM d'haute définition qui étaient victimes d´un traumatisme dans les 100% des cas. Ces résultats sont superposables à ceux rapportés par Kouakou [1] en Côte d´Ivoire et Barreau [3] en France et Hiroual [4] au Maroc. Mais Saraiya [5] trouvait dans la même situation un pourcentage faible de fractures du rocher soit 41%. Pour Darrouzet [6] en France, les étiologies des fractures du rocher étaient dominées par les accidents de sport suivis des plaies par arme à feu et non par les traumatismes post AVP. Cela peut s´expliquer par le respect du code de la route en France contrairement au Maroc où on observe moins de respect du code de la route malgré un bon état des routes. Il existe d´autres étiologies des traumatismes du rocher notamment les causes pénétrantes par CVB par arme à feu, le barotraumatisme et les causes thermiques [7]. On a retrouvé également des causes par chute qui est rare (2 cas seulement sur 12 cas dans notre série). Les signes cliniques étaient dominés par l´otorragie (50% des cas) suivie de la paralysie faciale (25% des cas) dont les vertiges accompagnaient ses signes dans 5 cas environs. Les notions de nausées, vomissement et de trouble de l´équilibre ont été retrouvés dans notre série. Hiroual a retrouvé dans son étude que tous les patients avaient une otorragie et (52%) une paralysie faciale, mais la notion de vertige n´a pas été abordée précisément dans ses études [4].

Tous les types de fracture ont été observés dans notre série (100% des cas). Ces lésions siégeaient majoritairement à gauche ce qui est superposable à la littérature en France [8, 9], en Afrique plus précisément en Côte d'Ivoire les lésions siègent à droite [1]. Ces types de fracture sont détaillés selon les différents types de classification [10]. Il existe plusieurs types de classifications dont celle de Ramadier et Causse (qui décrivent les fractures selon le plan transversal ou longitudinal) retrouvés dans 66,7% dans notre série superposable dans la littérature [10]. La classification de Aubry et Pialoux (qui a leur tour décrivaient les fractures selon l´atteinte labyrinthiques) [10]. L´atteinte de la chaine ossiculaire représente plus de 25% des fractures du rocher qu´il soit un trait de fracture ou d´une luxation ossiculaire. L´atteinte ossiculaire représentait 41,6% dans notre série légèrement inférieure à la littérature dont 26,67%, 28,95%, 30% respectivement rapportés par Sonhayé, Hiroual et Meriot [4, 10-12]. Les lésions du canal facial se manifestent par une paralysie faciale dans 50% des cas. On a retrouvé 1 cas d´atteinte du nerf facial dans notre série déceler par la TDM du rocher, mais l´IRM reste la technique d´imagerie de référence en mettant en évidence un aspect épaissi et une prise de contraste après injection de gadolinium sur le trajet du VII et un hématome du ganglion géniculéumalléus et de l´incus [1, 13]. Les lésions vasculaires comme la dissection de la carotide interne et la survenue d´un accident vasculaire cérébral (AVC) sont les principales causes en cas de traumatisme des rochers. Il y a aussi le risque de thrombophlébite en cas d´atteinte des sinus veineux [1, 8, 14]. Un cas d´atteinte du canal carotidien a retrouvé dans notre étude. On n´a pas retrouvé des cas d´AVC ou de signe de thrombophlébite dans notre série. La fistule périlymphatique n´a pas été retrouvée dans notre série. Mais l´IRM est examen de référence pour la détection exacte de la fistule [1, 13, 14].

## Conclusion

La tomodensitométrie permet d'affirmer l'existence de la fracture, de décrire l'orientation du trait et de préciser les différentes structures atteintes. Elle peut être réalisée à distance devant la persistance d'une surdité de transmission ou de perception, une paralysie faciale secondaire, des vertiges. L'IRM n'est réalisée qu'en seconde intention si les symptômes restent inexpliqués ou en cas d'anomalie impliquant les structures intracrâniennes.

### Etat des connaissances sur le sujet

- *La difficulté d´interprétation du scanner du rocher dans le cadre du traumatisme direct du rocher ou dans le traumatisme cranio-encéphalique du fait de la diversité des formes cliniques et de la complexité de l´anatomie de l´oreille*.

### Contribution de notre étude à la connaissance

- *C´est la confirmation et la pertinence du rôle essentiel du radiologue et la place du scanner hélicoïdal dans le bilan lésionnel avec une description minutieuse des structures atteintes et les lésions associées en fin d´évaluer des complications potentielles et des séquelles*.

## Remerciements

Nos remerciements à tous les personnels du département de radiologie du CHU Ibn Rochd de Casablanca- Maroc et les collaborateurs de la Clinique Médicale Marie Curie de Bamako-Mali.
